# Intervening infant and young child feeding among Indian tribes – a scoping review

**DOI:** 10.1186/s12889-026-26560-9

**Published:** 2026-02-11

**Authors:** Rithu Sathiyamoorthy, Anusree Prabhakaran, Divya Sussana Patil, Arathi P. Rao

**Affiliations:** 1 Department of Global Public Health Policy and Governance, Prasanna School of Public Health, Manipal Academy of Higher Education, Manipal, Karnataka 576104 India; 2 Department of Health Technology and Informatics, Prasanna School of Public Health, Manipal Academy of Higher Education, Manipal, Karnataka 576104 India; 3 Centre for Evidence-Informed Decision-Making, Prasanna School of Public Health, Manipal Academy of Higher Education, Manipal, Karnataka 576104 India

**Keywords:** Breastfeeding, Community participation, Complementary feeding, Health behaviour, Health promotion, Indigenous health, Infant feeding, Infant nutrition; nutritional intervention

## Abstract

**Background:**

In India, 40.2%, 23.1%, and 39.4% children below five years amongst the 104 million Scheduled Tribes (STs) are stunted, wasted, and underweight, respectively. Although optimal infant and young child feeding (IYCF) practices can substantially improve nutrition in early life, they are highly influenced by cultural beliefs, social exclusion, and sociodemography, notably in STs, necessitating culturally and contextually tailored interventions. This scoping review aimed to map the existing interventions designed to enhance IYCF among Indian STs.

**Methods:**

Search was conducted on PubMed (NCBI), Embase (Elsevier), Web of Science (Clarivate), and CINAHL (EBSCO) using search strings built and modified using keywords such as ‘India’, ‘tribe’, and ‘infant and young child feeding’. Google Scholar search and citation search were also conducted. The final search was conducted on 27 July 2024. Two-stage screening was conducted on Rayyan software, a screening tool.

**Result:**

Out of 1387 results retrieved, 26 articles were included in this review. Citation search and grey literature provided 22 interventions. Overall, interventions varied from traditional face-to-face counselling to the use of audiovisual aids, such as videos and short films. They were delivered at various levels, including individual, interpersonal, organizational, and community. Mothers, fathers, grandmothers, and community members were targeted with holistic interventions that delivered key messages not only on IYCF in silos, but also on education, rights, marriage, reproductive health, maternal health, water, hygiene, sanitation, and agriculture. Interventions often focused on community participation, responsive interventions, and intersectoral convergence.

**Conclusion:**

This review revealed that interventions aimed at improving IYCF among Indian STs predominantly employed a holistic approach, fostered community participation, leveraged existing local platforms and utilized linguistically tailored audiovisual aids. However, a few gaps persisted in active family involvement, motivation and incentivization of community members to serve as facilitators, and regular attendance of the target population during implementation. This highlights the need to focus on sustaining these interventions and scaling them.

**Supplementary Information:**

The online version contains supplementary material available at 10.1186/s12889-026-26560-9.

## Background

The first 1000 days of life, including approximately 270 days in utero and the first two years postnatally, constitute a critical window for implementing effective nutritional interventions. Optimal infant and young child feeding (IYCF) practices during this period are fundamental to ensuring healthy growth, development, and long-term health outcomes. Core components of IYCF include the initiation of breastfeeding within the first hour of birth, exclusive breastfeeding for the first six months, the timely introduction of age-appropriate complementary foods between six and eight months, and continued breastfeeding up to two years of age or beyond [1].

These practices have a profound impact on growth, development, nutritional status, health outcomes, and overall survival of a child through adolescence into adulthood. Adherence to optimal IYCF practices can reduce neonatal mortality by 20% and under-five mortality by 13%, largely due to the protective effects of early and exclusive breastfeeding against infections such as diarrhoea and pneumonia, which are among the leading causes of death of children under five years of age [2]. Adequate nutrition during infancy and early childhood supports healthy physical growth, brain development, and the achievement of a child’s full genetic potential. They also have lasting effects, reducing the risk of chronic diseases such as obesity, diabetes, and cardiovascular disorders later in life. Proper feeding practices help prevent both undernutrition and micronutrient deficiencies, which largely contribute to stunting, cognitive impairment, and increased susceptibility to disease. Breastfeeding also benefits mothers by reducing the risk of postpartum haemorrhage, ovarian, uterine, and breast cancers, and by supporting birth spacing [3].

To address the significance of optimal IYCF practices, WHO and UNICEF introduced the Global Strategy for Infant and Young Child Feeding in 2003 [4]. Later, in 2012, the World Health Assembly adopted Resolution 65.6, setting a target to increase the prevalence of exclusive breastfeeding to at least 50% by 2025. Additionally, the Sustainable Development Goal (SDG) 2 seeks to eliminate hunger and malnutrition by 2030 through universal access to safe, nutritious, and sufficient food [5].

Despite the above-mentioned efforts, achieving widespread adherence to optimal IYCF practices remains a significant public health challenge in low- and middle-income countries like India, where the prevalence of child malnutrition is high. According to National Family Health Survey (NFHS) – 5 conducted between 2019 and 2021, 35.5%, 19.3%, and 32.1% of the Indian children under five years were stunted, wasted, and underweight respectively. However, only 63.7% were exclusively breastfed, 45.9% were introduced to complementary food at 6–8 months, and 11.3% of them were fed a minimally acceptable diet at 6–23 months [6]. This gap is particularly high in marginalized communities. Home to the world’s second-largest tribal population, India has 104 million tribal people called Scheduled Tribes (STs), accounting 8.9% of the national population, across 705 officially recognized ST groups [7]. ST encompasses India’s indigenous communities, which have distinct languages and cultural practices, are economically disadvantaged, geographically isolated, and hesitant or shy to engage with the broader Indian society [8]. A comparison between NFHS-4 conducted in 2015–2016 and NFHS-5 conducted in 2019–2021 indicated improvements in several health and nutrition indicators among ST populations; however, the burden of child undernutrition persists. Among ST children under five years of age, 40.2% were stunted, 23.1% were wasted, and 39.4% were underweight. While 71.2% of the ST infants below six months were exclusively breastfed, only 46.6% were timely breastfed within the first hour of birth, and 11.2% aged 6–23 months received an adequate diet [9].

Recognizing this gap, the Government of India, over five decades, has implemented a range of policies and programs aimed at promoting IYCF and addressing malnutrition. The Integrated Child Development Services (ICDS) scheme, initiated in 1975, is a flagship initiative that provides supplementary nutrition, growth monitoring, and nutrition counselling to pregnant and lactating mothers and children under six years, primarily through Anganwadi centres and Anganwadi workers (AWW) across the country. Subsequent programs, including Reproductive and Child Health (RCH) I and II, the Reproductive, Maternal, Newborn, Child and Adolescent Health (RMNCH + A) strategy launched in 2013, further emphasized the importance of optimal IYCF practices [10–12]. One of the key initiatives is the Mother’s Absolute Affection (MAA) program, introduced in 2016 as a nationwide effort to promote breastfeeding through awareness campaigns, capacity building of healthcare workers, and community engagement. This programme emphasizes breastfeeding for the first six months and continued breastfeeding along with appropriate complementary feeding [10].

The POSHAN Abhiyaan or the Prime Minister’s Overarching Scheme for Holistic Nourishment is a comprehensive nutrition mission, introduced in 2018, aimed at reducing stunting, undernutrition, and low birth weight by promoting behavioural change communication and improving service delivery related to maternal and child nutrition [13]. The Home-Based Care for Young Children (HBYC) initiative extends community-based support through Accredited Social Health Activists (ASHAs), focusing on counselling and follow-up for optimal feeding practices [14].

Despite the existence of numerous initiatives, it is crucial to understand that STs have distinct child-rearing practices that are deeply influenced by their communal way of living, tradition-based approaches, and environmental and socioeconomic characteristics, which are different from non-STs. Addressing the nutrition-related challenges in these populations necessitates more culturally sensitive and evidence-based interventions compared to non-STs. Tailored strategies that respect their distinct practices while promoting optimal and sustainable improvements in child health and nutrition are vital for reducing health disparities among ST communities and progressing towards SDG 2.

This study is a predecessor of a primary study aimed at developing an intervention to enhance IYCF practices among Indian STs. A scoping review methodology was selected to help map the current evidence on interventions available. A preliminary search of PubMed (NCBI), the Cochrane Database of Systematic Reviews and *JBI Evidence Synthesis* revealed no existing or ongoing reviews specifically addressing this topic.

Hence, the objective of this scoping review was to *map the various interventions delivered to improve IYCF among Indian ST communities.*

## Methodology

This scoping review was conducted in accordance with the JBI methodology for scoping reviews [15] and reported as per the Preferred Reporting Items for Systematic Reviews and Meta-Analyses (PRISMA) guidelines adapted for scoping reviews (Additional file 1) [16]. The protocol was registered with the Open Science Framework (10.17605/OSF.IO/G6VST).

Review question: What are the interventions for enhancing infant and young child feeding among Indian ST populations?

### Search strategy

A comprehensive search strategy was developed using index terms and keywords relevant to the population, concept, and context of this review. The search was tailored for databases such as PubMed (NCBI), Embase (Elsevier), Web of Science (Clarivate), and CINAHL (EBSCO). The final search was conducted on 27 July 2024. The full search strategy is available as Additional file* 2*. The reference lists of reviews were screened for additional studies. Supplementary search was conducted in Google Scholar, and grey literature was identified through a manual search of the ICDS websites of all Indian states.

### Study/source of evidence selection

**Fig. 1:**
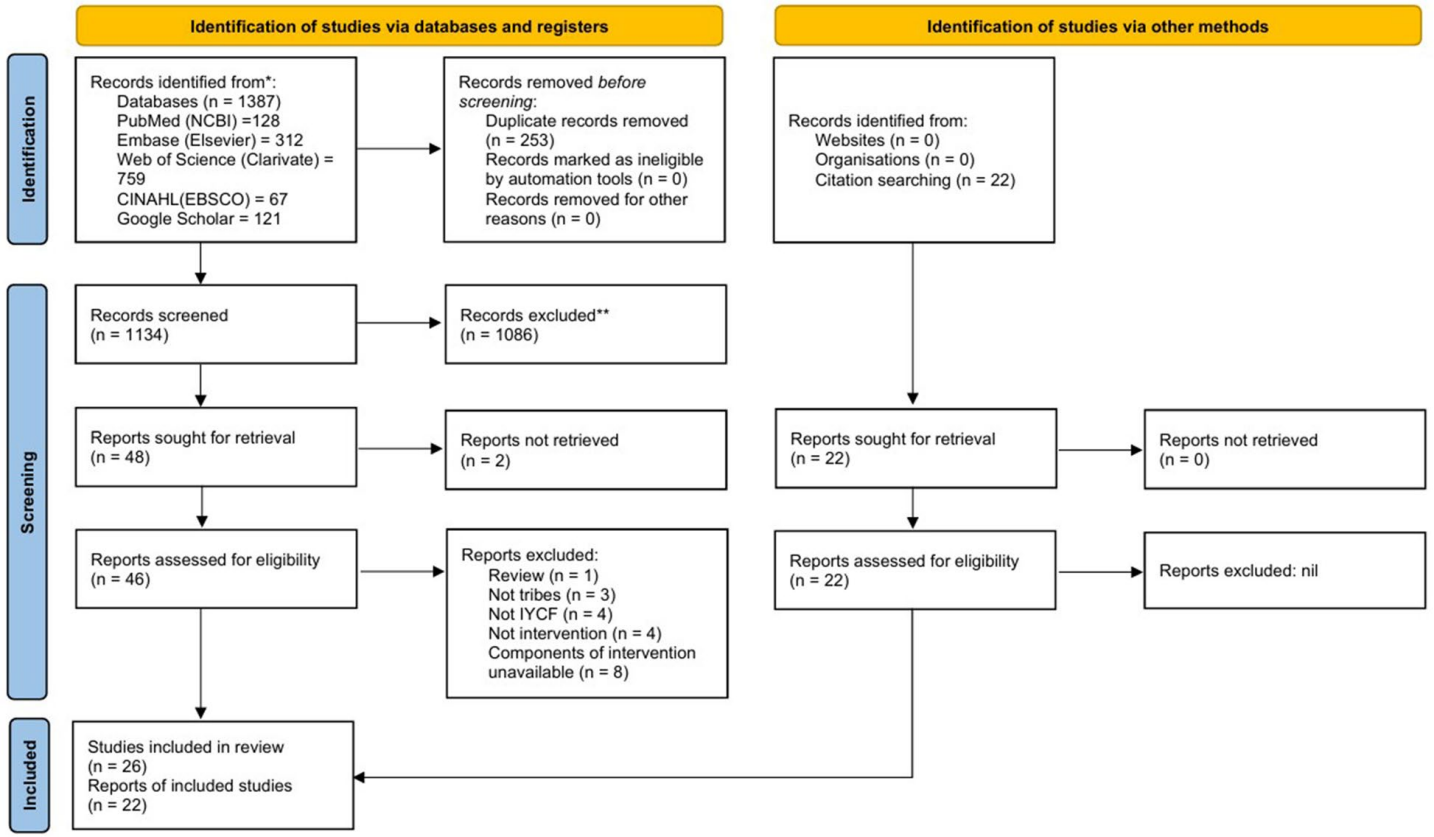
PRSIMA flow diagram depicting the screening process

The results from each database were extracted into Rayyan software, a screening tool, for deduplication and screening of articles [17]. A two-stage screening process, comprising title and abstract screening followed by full-text screening of the articles, was independently conducted by two reviewers (RS and AP) based on the eligibility criteria (depicted in Table 1), and conflicts were resolved through discussion with two additional reviewers (DSP and APR). The screening process is illustrated using the PRISMA flowchart in Fig. 1.

**Table 1:** Eligibility criteria of included studies

Population	ST population *Inclusion Criteria:* Only studies involving Indian STs were included
Concept	Intervention aimed at enhancing IYCF *Inclusion Criteria:* Only interventional studies that have studied IYCF indicators as outcomes were included. These indicators included the practices: timely initiation of breastfeeding, feeding of colostrum, early initiation of breastfeeding, exclusive breastfeeding up to six months of age, introducing complementary food at six months along with breastmilk, continuation of breastfeeding up to two years of age, minimal dietary diversity, minimum acceptable diet, bottle feeding, introduction of water, minimum food frequency, food accessibility, food affordability, food availability, food taboo, weaning practices, feeding of junk food, traditional feeding practices, formula feeding, wet nursing, donor breastmilk; and the knowledge and attitude of caregivers towards these practices
Context	India *Inclusion Criteria:* Only studies involving STs in the Indian context were included
Study design	*Inclusion Criteria:* Descriptive and experimental study designs that explained the components of the interventions were included *Exclusion Criteria:* All types of reviews, text, conference abstracts, book chapters, and opinion papers were excluded
Language	Only studies published in English were included
Publication date	The publication date of articles was not limited

### Data extraction

Data was extracted independently by two reviewers (RS and AP) in a pre-designed Microsoft Excel spreadsheet. The data extraction sheet included the author and year of publication, study setting, study population, intervention delivered at different levels, components of the intervention, and key information delivered. It was pilot-tested by RS and AP on a sample of 10 studies, and modifications were made based on feedback from DSP and APR to ensure consistency and resolve conflicts in data extraction.

The extracted data was summarized descriptively. The key components of the interventions are graphically presented.

## Results

A total of 1266 results were retrieved by conducting the search on PubMed (*n* = 128), Embase (*n* = 312), Web of Science (*n* = 759), and CINAHL (*n* = 67). Additionally, a search was also conducted on Google Scholar, which provided 121 articles. Hence, a total of 1387 articles were retrieved and uploaded to Rayyan software. Of them, 253 duplicates were removed, and two-stage screening of 1134 articles was conducted independently by RS and AP based on the eligibility criteria. Out of these, 1086 articles were excluded based on title and abstract. The full text of 46 articles retrieved out of 48 was screened and 20 of them were excluded for the following reasons: one was a review [18], three articles did not include ST populations [19–21], four articles did not discuss IYCF [22–25], four articles did not discuss any intervention [26–29], and eight articles did not explain the components of the intervention [30–37]. Hence, a total of 26 articles were included from the database search.

Citation search was conducted from the bibliography of the excluded reviews, which provided 22 interventions, of which six, namely, Nirantar [38], Ankur [39], Home-Based Neonatal Care [40], Mitanin [41], PANChSHEEL [42], and a phone-based intervention [43] exclusively focused on ST populations. Also, a few interventions of the ICDS and POSHAN Abhiyaan were considered in this review, for which STs were a part of the targeted population, namely, Anchal Se Angan Tak, Baby-Friendly Community Health Initiative, Dulaar, Integrated Nutrition and Health Programme, Rajmata Jijau Mother–Child Health and Nutrition Mission, Tamil Nadu Integrated Nutrition Programme, and Village Health and Nutrition Days (including Fixed Nutrition and Health Days) [44], Nutri-Garden, Poshan Doot [45], Keno Parbo Na [46], Spotlight [47], Vistaar [48], and Swasthya Saathi [38]. The screening process has been depicted in the PRISMA flow diagram in Fig. 1.

The characteristics of the included studies have been depicted in Additional file 3.

### Study setting

Few interventions were delivered exclusively within a single state like Andhra Pradesh [49, 50], Karnataka [51], Tamil Nadu [44], and Telangana [52] in South India; Chhattisgarh [41, 45] in Central India, Bihar [53–55], Jharkhand [56], Odisha [57], and West Bengal [46, 58–61] in East India; Maharashtra [39, 40, 43, 44, 47, 62, 63], Gujarat [38, 64–66], and Rajasthan [36, 38, 44, 45] in West India; and Uttar Pradesh [44, 67, 68] in North India. Few other interventions were delivered across multiple, not necessarily always neighbouring, states like in Madhya Pradesh and Uttar Pradesh [44], Uttar Pradesh and Jharkhand [48], Jharkhand and Bihar [44], Jharkhand and Odisha [69–72], Odisha, Gujarat, and Maharashtra [73], Chhattisgarh, Jharkhand, Madhya Pradesh, Odisha, and West Bengal [74], Andhra Pradesh, Bihar, Chhattisgarh, Madhya Pradesh, Odisha, Rajasthan, Uttar Pradesh, and West Bengal [44].

This geographic distribution of the interventions retrieved from database search indicates that interventions were often designed to encompass neighbouring states like Bihar, Jharkhand, Madhya Pradesh, Odisha, Uttar Pradesh, and West Bengal. In contrast, interventions targeting South Indian states like Andhra Pradesh, Karnataka, Tamil Nadu, and Telangana were designed in silos for each state.

### Types of study designs

Among the 26 studies retrieved from database search, three were descriptive studies [56, 59, 64]. Thirteen studies had adopted a quasi-experimental interventional design [50, 51, 54, 55, 60, 63, 65, 67, 68, 71–74], and ten were randomized controlled trials [49, 52, 53, 57, 58, 61, 62, 66, 69, 70].

### Target population

Women of reproductive age between 15–49 years [38, 69], including adolescents [59], pregnant women [40, 43–45, 49, 59, 63, 66, 70, 73], and mothers with children aged below five years [38, 39, 41, 44–47, 49–53, 55–60, 62, 63, 65–72, 74] were primarily targeted to receive the interventions aimed at improving IYCF among ST populations. Notably, only two of the above-mentioned interventions included women of reproductive age without any experience of pregnancy or childbirth [38, 59, 69] and only 10 out of 46 interventions included pregnant women [40, 43–45, 49, 59, 63, 66, 70, 73].

Family members [44, 64, 66, 73], particularly fathers [52, 56, 59], and grandmothers [38–40, 44, 47, 56, 59] were also targeted since as early as 1998 and 2002, respectively. This strongly suggests that, although other caregivers, such as fathers and grandmothers, were targeted in a few interventions, most of them exclusively (32 out of 46) focused on raising awareness and empowering mothers who were the primary caregivers in the community. Community members [46, 64, 70] including matri sahayak gut/maternal support group(MSG) [45], local women’s self-help group (SHG) members [54, 55, 61], community leaders [59, 64], community influencers [47], panchayat members [59], and local school teachers [59] were targeted. Community health workers (CHWs) like ASHA and AWW [62] and were also included.

### Facilitators

Several stakeholders played the role of facilitators in delivering these interventions. Female volunteers from the community were trained to deliver the interventions. Given the country’s cultural and linguistic diversity, these female volunteers were designated different titles across different regions. They were referred to as Saheli [54], Poshan Sakhi [74], Su-Poshan Karyakarta [70], Swasthya Sakhi [67], Poshan Doot [45], Swasthya Saathi, community health educator [38], community champion [36], village health workers [38–40], merely as a community volunteer [41], change agent, or local resource person [44] in a few studies. These women were either mothers [44, 49, 63, 71] or members of local women’s groups/SHGs/Mahila Mandals [53, 56, 57], merely female members of the community [54, 55, 61, 67, 69, 70, 72, 74] or leaders of women’s working groups [44]. Community leaders like panchayat members [44, 70], local teachers [44], CHWs like ASHA, AWW, Auxiliary Nurse Midwives, Trained Birth Attendants [38, 39, 43, 44, 47, 52, 60, 64, 66, 68, 70, 73], health workers [52, 56, 66, 70], physicians [59], ayurvedic doctors [43], lactation counsellors [43], and nutritionists [59] also delivered the interventions. Few interventions were delivered by NGO workers, case workers, and research staff [39, 50, 51, 59, 60, 62, 65].

This demonstrates that women from the community, alongside healthcare workers and researchers, were actively involved in delivering the interventions. These women were either part of the local community-based organizations or were primary caregivers who were currently or previously involved in IYCF.

### Modes of delivery

Interventions have been implemented at various levels—individual, interpersonal, community, and organizational – constituting a multilevel approach.

#### Individual level

Twenty-four interventions comprised a component of home visits. The facilitators mentioned above visited the houses of the target population for face-to-face, one-on-one communication. In these home visits, they provided counselling to mothers and their families about various childcare and IYCF practices [38–41, 43, 44, 47–49, 51, 52, 56–59, 62–68, 70, 72]. In three studies, the facilitator demonstrated food preparation during these home visits [44, 49, 58]. They also sent short messages to the mothers about these feeding and cooking practices in two studies [43, 62].

#### Group level

Twenty-two interventions had a component of delivery at a group level. Counselling was provided in group meetings in two studies [50, 73] or in a child nutrition centre in one intervention [44]. In six of the 22 studies mentioned previously, the intervention was delivered in schools [36] and at SHG meetings in the form of dissemination of information [67], counselling [44, 46, 55], and participatory and interactive group discussions [19, 36, 39, 44, 46, 53, 54, 57, 74]. Participatory learning action cycle was adopted in five studies [57, 69–72]. Case studies were discussed in one study [69], while cooking demonstrations were conducted in group level in four studies [44, 59, 62, 70, 72]. Interactive storytelling was a part of these group-level activities in four studies [69, 70, 72, 74]. Information was also disseminated through lectures [60, 62], role plays [62, 69, 72], and games [59, 69, 70, 72].

While existing platforms like SHG have been extensively utilised by the interventions, it has also been reported that attendance in the SHG meetings was irregular and needed to be addressed [55, 74].

#### Community level

Nineteen interventions had a community-level component. These events included village meetings, village level or community meetings [64, 67, 73], health camps [38, 56, 74], cooking demonstrations [44, 45, 53, 74], sharing experiences of cooking and feeding [53], recipe contest [45], counselling, celebration of grandmothers, world breastfeeding week, rallies [56], poshan rath [45] [52], village health and nutrition day [44, 48, 73], godhbarai (baby shower), janamdin (birthday celebration) [44], annaprasan diwas (introduction of solid food) [44, 67] and felicitation of role model mothers [53].

Community participation has been emphasized in several studies, since as early as 2005 and especially between 2012 and 2020. Such interventions were more frequently delivered in Odisha than in other states [69–71]. These interventions, with women from the community as key facilitators, employed participatory approaches that demanded community mobilization and engagement. However, proper engagement and participation of these stakeholders was reported to be limited and recommended to be encouraged [53, 54, 57, 60, 69–74].

However, this study revealed that only four interventions had delivery components at all three levels [44, 57, 62, 67].

**Fig. 2:**
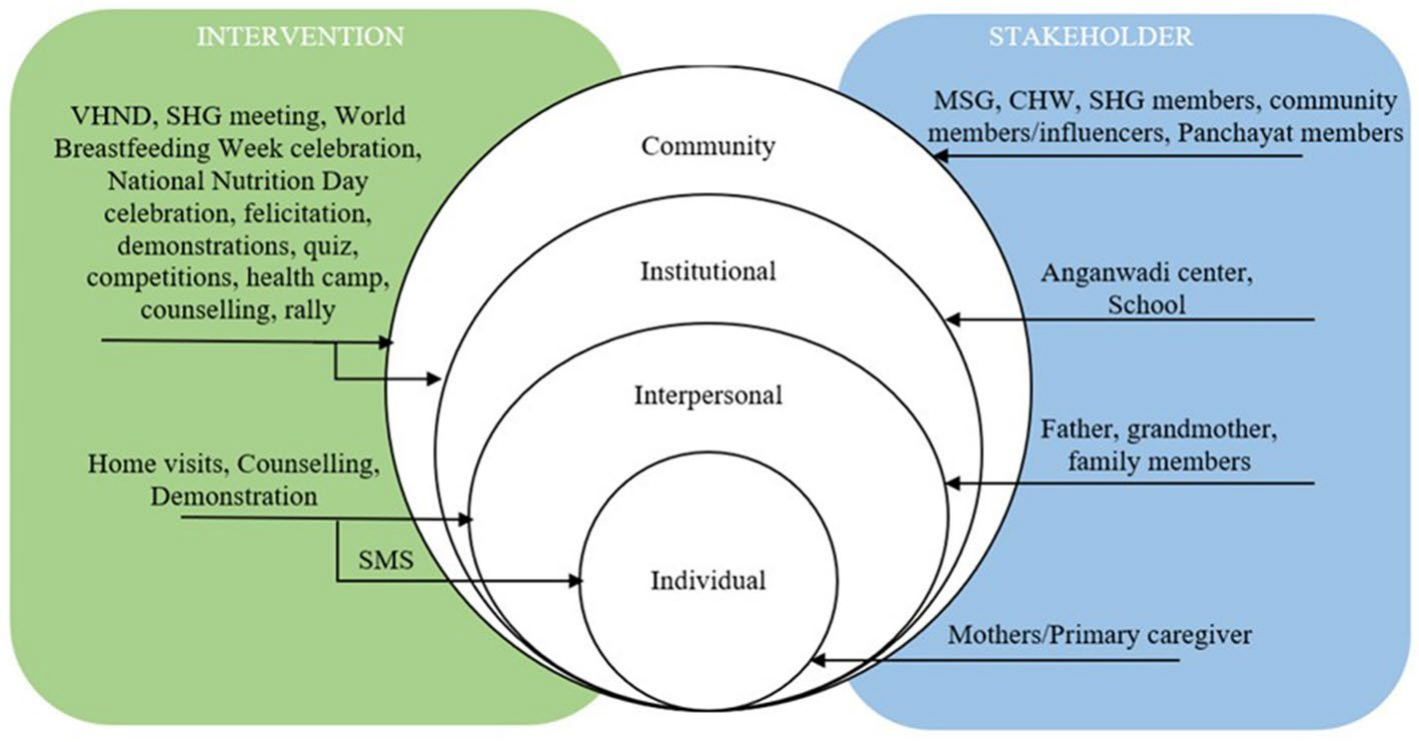
Framework depicting – across levels – the strategies and stakeholders involved in the interventions delivered to improve IYCF among Indian STs

Figure 2 depicts the various strategies implemented in the interventions, and stakeholder involvement across different levels of the social ecological model.

### Aids for delivery of intervention

Visual aids like banners [60], posters [59, 73], pictorial posters [64], books [59], flipcharts [50, 64, 73], pictorial flipcharts [49], picture cards [69–72], wall writings [56], leaflets [65], overlay charts, flannelgraphs, window charts, projection charts, flash cards, magnetic cards and stick puppets [50] were used in a few studies to disseminate the key messages. Audio aids like tapes [59] and songs [56] were used in a few studies. Several studies employed the use of audiovisual aids like videos [41, 53, 59, 60, 66, 67, 73], participatory videos [57], short films [65], folk media like puppet shows [45, 56], local folk dance [56], nukkad natak/street play and kala jattha/street theatre [45].

All these aids were delivered in the local language to ensure comprehensibility and retention of information by the target population. Although audiovisual aids have been employed since as early as 2002, they were frequently delivered only in certain states, such as Maharashtra, Gujarat, and West Bengal.

### Key messages delivered

The core messages delivered in these interventions aiming at improving IYCF among STs were related to breastfeeding practices, complementary feeding practices, and child nutrition.

Among breastfeeding practices, timely initiation of breastfeeding, avoiding prelacteal feeding, importance of colostrum, proper latching technique, position for feeding, responsive feeding, hunger cues, exclusive breastfeeding, its duration and frequency, lactation issues, sustained breastfeeding, continuing breastfeeding along with complementary food, and avoiding bottles were emphasized [36, 40, 41, 43–51, 53–56, 59, 60, 62, 63, 65–69, 71–74].

Key information about complementary feeding like timely initiation of complementary food, importance of complementary feeding, age-appropriate food, consistency of food, frequency of feeding, food recipes based on locally available and affordable food items, kitchen garden, dietary diversity, role of different food groups, preserving nutrients while cooking, cooking fuel, food enrichment, weaning, and supplementary nutrition [36, 38, 41, 43–47, 49–51, 53–56, 58–62, 65, 66, 68, 70–72, 74].

Seven among these interventions discussed only about breastfeeding and not about complementary feeding [40, 44, 59, 63, 67, 69, 73]. These studies primarily targeted pregnant women and recent mothers. One study had spoken only about complementary feeding and did not have information on breastfeeding [61].

Key information on growth promotion, malnutrition, and the life-cycle approach/intergenerational cycle of malnutrition was also delivered [39, 40, 44, 45, 48, 70, 74].

In addition to these core messages on IYCF and nutrition, further information was provided on related factors, including reproductive health, maternal health, newborn care, hygiene practices, and empowerment.

Reproductive health messages on safe sex practices, family planning, birth spacing, and small family norm were delivered [38, 44, 47, 50, 52, 56, 59, 67, 70]. Key information about maternal health, like pregnancy, nutrition, and its importance during pregnancy, consumption of iron, folic acid, iodine, and calcium during pregnancy, and identification of danger signs during pregnancy, was delivered [38, 40, 43, 44, 46, 47, 50, 52, 55–57, 59, 62, 67, 70, 74]. Information on safe motherhood practices, like birth preparedness and safe-delivery practices, was also delivered [44, 52, 54, 55, 63, 64, 67, 69].

Despite been provided, only two out of ten interventions that discussed about reproductive health had targeted men along with women [52, 56, 59] and only three involved couples or women with no prior experience of pregnancy or childbirth [38, 59].

Key messages on newborn care practices like cord care, warmth, skin-to-skin contact, delayed bathing, identification of newborn danger signs, and hygiene practices for the prevention of infection were provided [38–40, 44, 48, 50, 54, 59, 64, 67, 71, 73]. Childcare practices like proper sleep, play, immunization, deworming, prevention of illness, identification of childhood illness, management of diarrhoea, child morbidity, habit training, gender-sensitive childcare, and pre-school informal education were emphasized [36, 38, 44, 46, 47, 50, 52, 54, 56, 57, 59, 66, 70–72, 74]. Concepts like roles of fathers and grandmothers and family cohesion were also emphasized [52, 59].

Hygiene practices like safe water storage, sanitation, use of toilets, waste management, handwashing, personal hygiene, feeding hygiene, environmental hygiene, and use of bed nets were also emphasized [36, 38, 46, 49, 50, 54, 55, 59, 60, 70, 72–74].

Information about gender equality [50], gender issues, leadership, income generation [44], entitlements to rights, women’s agency [70, 74], entitlements to the public distribution system [41], marriage [50, 52, 74], and nutrition-sensitive agricultural practices [57] were delivered.

It is also notable that the studies have employed a tailored approach, where the interventions were informed by a formative phase, which ensured sensitivity to the culture, beliefs, acceptability, food affordability, and availability of the target population. However, only 10 studies had holistically included information on IYCF alongside those on nutrition-specific and nutrition-sensitive determinants [36, 50, 54, 55, 59, 60, 70, 73–75].

## Discussion

**Fig. 3:**
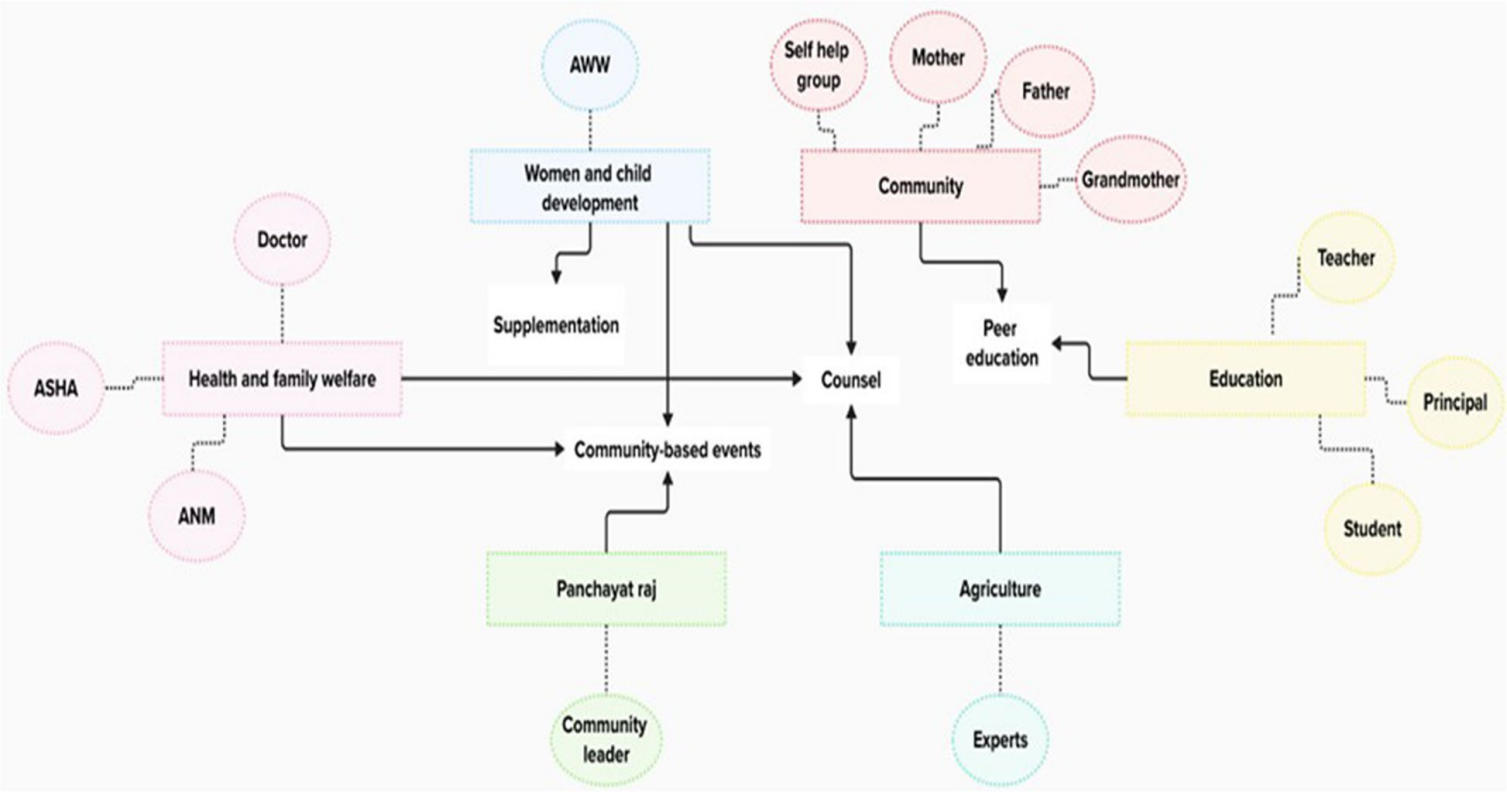
Convergence of various ministries to improve IYCF

IYCF in India – diverse and deeply rooted in cultural norms and beliefs—has been primarily governed by the Ministry of Women and Child Development for over five decades, with collaborative efforts across multiple ministries [76] as depicted in Fig. 3. The influence of culture on IYCF is pronounced among the ST population, whose distinct livelihood, education, and socioeconomic status differentiate them from the non-ST population and shape their IYCF [77, 78]. Hence, promoting these practices among ST populations is crucial, necessitating culturally sensitive and contextually feasible interventions. In addition to efforts undertaken by ICDS, several interventions targeting IYCF among ST populations have emerged from academic research or as projects by non-governmental organizations.

This review mapped the interventions delivered in India that aimed to improve IYCF practices among ST populations. It revealed that most interventions targeted mothers, but were also family- and community-oriented, culturally and linguistically tailored, holistic, multi-faceted, with several adopting community-based participatory approaches.

These interventions were frequently delivered in a few states. The proportion of ST population to the state population for these states was Andhra Pradesh (7%), Bihar (1.28%), Gujarat (14.75%), Jharkhand (26.21%), Karnataka (6.95%), Madhya Pradesh (21.09%), Maharashtra (9.35%), Odisha (22.85%), Telangana (9.08%), Uttar Pradesh (0.57%), Uttarakhand (2.9%), or West Bengal (5.8%) [79, 80]. This revealed a substantial evidence gap in the literature in states with high ST representation, like Mizoram (94.44%), Nagaland (86.46%), and Meghalaya (87.6%). This underrepresentation may be attributed to the higher literacy rates among women of reproductive age in these states- Mizoram (94%), Nagaland (83.4%), Meghalaya (74.5%), and Kerala (97.4%), which potentially influences their health behaviour and access to information differently compared to other ST regions, reducing the perceived need for interventions. The geographic distribution of the interventions also indicates a notable research gap in South India and Northeast India. This disparity might be attributed to challenges in accessing ST populations in these predominantly hilly and difficult-to-reach areas, in contrast to relatively flat and more accessible areas of Central India [81].

Literature reveals that pregnant Indian women had poor knowledge of IYCF, and their exclusive breastfeeding practices and self-efficacy improved on receiving education on these practices [82–84]. Hence, targeting pregnant women along with mothers with children to receive IYCF interventions would ensure better knowledge on IYCF, decision-making for IYCF and self-efficacy postpartum, particularly in egalitarian ST communities in India, where women have equal rights and freedom like their male counterparts [85]. However, the current review revealed that while mothers with children below two years were primarily targeted to receive the interventions delivered to improve IYCF, only two included women of reproductive age with no previous experience of pregnancy or childbirth and 10 interventions targeted pregnant women to ensure their readiness for IYCF immediately after childbirth. Additionally, not all studies provided information on both breastfeeding and complementary feeding. This is considered inadequate, as studies conducted in Nepal in 2020 and Turkey in 2022 revealed that mothers with less knowledge about complementary feeding also initiated solid food earlier than six months, which affected exclusive breastfeeding [86, 87].

While most interventions emphasized IYCF along with neonatal and childcare, a few were more holistic with a life cycle approach. They provided information about education, leadership, rights, gender equality, marriage, reproductive health, pregnancy, antenatal and postnatal health, maternal and child nutrition, water, sanitation, and hygiene. Such a holistic approach is considered to support mothers by empowering them in terms of leadership, education, employment, and autonomy, which, when integrated with nutrition information, improved their knowledge on IYCF and collectively influenced child health outcomes [88, 89]. This review, however, identified a gap in holistic interventions aimed at improving IYCF among STs, which is particularly consequential in this population due to their sociodemographic characteristics and socioeconomic status.

The current review also revealed the low involvement of fathers in the interventions that discussed the significance of reproductive health like safe sex practices, birth spacing, and family planning. This is a particularly relevant finding since an interventional study conducted in rural India in 2016 showed that, when men were engaged in family planning counselling alone and along with their spouses, improved contraceptive practices [90]. Despite the assertion of active engagement of men in reproductive health and family planning and the 2000 National Population Policy of India, these stigmatized concepts are women-focused, though men are major decision makers [91–94].

When it comes to discussing about IYCF, the current review revealed that very few studies targeted fathers, grandmothers and other family members, depicting a gap in recognizing the significance of a secondary/non-primary caregiver in IYCF. Literature suggests that IYCF practices were better among mothers who received family and social support and were self-efficacious for breastfeeding [95, 96]. Especially among ST mothers, it was found that IYCF practices were better in joint family households [97]. This highlights the significance of sensitising family members about IYCF. In contrast, a study conducted in Uganda in 2024 revealed concerns of grandmothers influencing mothers with food-based beliefs [98].

Community participation is a repeatedly emphasized concept in the interventions included in this review. Many interventions employed participatory approaches, particularly the Participatory Learning and Action Cycle. The targeted mothers were encouraged to partake in group discussions where they discussed the challenges they face with feeding their child, and they were also motivated to develop solutions for these challenges through these effective interactions. This helped build autonomy on IYCF practices. These discussions also involved senior women from the community, who provide insights and solutions based on their similar experiences, to ensure their cultural sensitivity, acceptability, feasibility, and sustainability of these solutions. These women, when involved in all the steps of development of the intervention, had more ownership, respect, and reciprocated well to the intervention, making it sustainable [99]. Community-based peer support, and peer counselling, especially in low- and middle-income countries improved IYCF practices [100, 101].

Several studies included in this review also involved the local women’s groups/SHGs, who either received the intervention in a few studies or acted as facilitators in a few others. SHGs are voluntary collectives of women with similar economic backgrounds that foster women’s agency, empowerment, entrepreneurship, and financial inclusion [102]. Members of these groups are, evidently, mothers in the community or senior women who might be grandmothers. Community mobilization through participatory women’s groups like SHGs are known effective community models to improve child nutrition and health indicators [103, 104]. However, it is crucial to understand the challenges in community-based participatory research, especially among STs, like sustainability due to funding and participation of facilitators [105].

The information on IYCF was delivered using various audio, visual, and audiovisual aids to encourage interactive discussions and facilitate retention. These aids were culturally and linguistically tailored according to the target population. This contextualization improves the sense of inclusivity in the development of the intervention, acceptability and comprehensibility of the intervention, and resilience of the mothers in sustaining them [106, 107]. While a study conducted in India in 2020 showed that culturally appropriate educational intervention improved the infant feeding practices among vulnerable populations [108], a review revealed that this improvement in dietary practices was not attributed to cultural adaptation of interventions in ST populations [109].

This review revealed that key messages were delivered through various approaches, at an interpersonal level through home visits, at the community level through group discussions and community events, and at an organizational level through anganwadis and schools. While literature shows that intensive interventions, delivered through multiple platforms, are feasible and effective when compared with non-intensive interventions [110], only four out of the 46 included interventions incorporated strategies in all three levels.

Along with delivery of information, model mothers were recognized to motivate them to optimally feed their child and grandmothers were celebrated to motivate them to support the mothers. Celebrations like the initiation of complementary food and breastfeeding week emphasized to reinforce the importance of IYCF. Sensitising and motivating all stakeholders involved with IYCF is significant for socially cohesive communities [111, 112].

*Strengths*: This scoping review contributes to the limited existing literature on interventions aimed at improving IYCF practices among ST populations in India, considering they comprise 8.6% of the country’s population and have diverse cultural norms and beliefs. This review employed a comprehensive search strategy across four different databases, ensuring the inclusion of a substantial number of articles and grey literature.

### Limitations

This scoping review is not comprehensive since government reports could not be accessed.

## Conclusion

Inhabiting about 104 million STs, India reflects substantial cultural and socio-linguistic diversity. These populations differ significantly from non-ST populations in terms of cultural practices, belief systems, socioeconomic status, sociodemographic characteristics, all of which influence their health- and nutrition-related outcomes. Designing and delivering interventions to improve these outcomes requires a highly sensitive, context-specific and methodologically rigorous approach. Particularly, interventions aiming to improve their IYCF practices, which are largely influenced by culture and beliefs, must focus on nutrition-sensitive and nutrition-specific factors. This scoping review reveals that the interventions delivered among Indian STs to improve IYCF practices were mostly focused on mothers, sometimes family- and community-centred, culturally and linguistically tailored, holistic, and multi-faceted. Yet, gaps like the motivation and incentivizing of community members to facilitate the delivery of interventions and the regular attendance of the target population in the intervention must be addressed. Inclusion of fathers in discussions related to reproductive and maternal health, targeting pregnant women and prospective mothers in promoting IYCF, and delivering the interventions at all levels of the community through community-based participatory methods warrants focus. Emphasis must be given to make these interventions scalable and sustainable.

## Supplementary Information

Additional file 1: PRISMA-ScR checklist

Additional file 2: Search strings adapted for different databases

Additional file 3: Characteristics of included studies

## Data Availability

No datasets were generated or analysed during the current study.

## Abbreviations

ASHA: Accredited Social Health Activist
AWW: Anganwadi Worker
CHW: Community Health Worker
HBYC: Home-Based Care for Young Children
ICDS: Integrated Child Development Services
IYCF: Infant and Young Child Feeding
MAA: Mother’s Absolute Affection
NFHS: National Family Health Survey
POSHAN: Prime Minister’s Overarching Scheme for Holistic Nourishment
PRISMA: Preferred Reporting Items for Systematic Reviews and Meta-Analyses
SDG: Sustainable Development Goal
ST: Scheduled Tribe
SHF: Self Help Group
UNICEF: United Nations Children’s Fund
WHO: World Health Organization

## References

[1] World Health Organization. Infant and young child feeding . 2025. Available from: https://www.who.int/news-room/fact-sheets/detail/infant-and-young-child-feeding

[2] Phukan D, Ranjan M, Dwivedi LK. Impact of timing of breastfeeding initiation on neonatal mortality in India. Int Breastfeed J. 2018;13(1):27. 10.1186/s13006-018-0162-0. 29988694 10.1186/s13006-018-0162-0 PMC6029033

[3] World Health Organization. The importance of infant and young child feeding and recommended practices. In: Infant and Young Child Feeding: Model Chapter for Textbooks for Medical Students and Allied Health Professionals . Geneva; 2009. Available from: https://www.ncbi.nlm.nih.gov/books/NBK148967/. Cited 2025 May 8. 23905206

[4] World Health Organization & United Nations Children’s Fund (UNICEF). Global strategy for infant and young child feeding . Geneva; 2003. Available from: https://www.who.int/publications/i/item/9241562218

[5] World Health Organization. Global nutrition targets 2025: policy brief series (WHO/NMH/NHD/14.2). . 2014. Available from: https://www.who.int/publications/i/item/WHO-NMH-NHD-14.2

[6] International Institute for Population Sciences (IIPS) and ICF. National Family Health Survey(NFHS-5), 2019–2021: India Volume I. . Mumbai; 2021. Available from: http://www.rchiips.org/nfhs. Cited 2025 Nov 24.

[7] Ministry of Tribal Affairs. Year End Review 2024: Ministry of Tribal Affairs . 2025. Available from: https://www.pib.gov.in/PressReleasePage.aspx?PRID=2090883. Cited 2025 Jun 20.

[8] Bullard EC. Scheduled Castes and Scheduled Tribes . EBSCO Knowledge Advantage. 2025. Available from: https://www.ebsco.com/research-starters/social-sciences-and-humanities/scheduled-castes-and-scheduled-tribes. Cited 2025 Dec 4.

[9] Subramanian S V., Joe W. Population, health and nutrition profile of the Scheduled Tribes in India: a comparative perspective, 2016–2021. The Lancet Regional Health - Southeast Asia . 2024;20:100266. Available from: https://www.thelancet.com/action/showFullText?pii=S2772368223001269. Cited 2025 May 8. 10.1016/j.lansea.2023.100266 PMC10794098 38234700

[10] Ministry of Health & Family Welfare: Government of India. National Health Mission: Mothers Absolute Affection .. Available from: https://nhm.gov.in/index1.php?lang=1&level=3&sublinkid=1450&lid=799. Cited 2025 May 8.

[11] Ministry of Health & Family Welfare: Government of India. National Health Mission: RMNCAH+N . Available from: https://nhm.gov.in/index1.php?lang=1&level=1&sublinkid=794&lid=168.

[12] Ministry of Health & Family Welfare: Government of India. Reproductive and Child Health (RCH) Portal . Available from: https://rch.mohfw.gov.in/RCH/about-rch.aspx.

[13] Ministry of Electronics & Information Technology: Government of India. POSHAN Abhiyaan - PM’s Overarching Scheme for Holistic Nourishment .. Available from: https://www.india.gov.in/spotlight/poshan-abhiyaan-pms-overarching-scheme-holistic-nourishment. Cited 2025 May 8.

[14] Ministry of Health & Family Welfare: Government of India. National Health Mission: Home-Based Care for Young Child Programme (HBYC) . Available from: https://www.nhm.gov.in/index4.php?lang=1&level=0&linkid=492&lid=761. Cited 2025 May 8.

[15] Peters MDJ, Marnie C, Tricco AC, Pollock D, Munn Z, Alexander L, et al. Updated methodological guidance for the conduct of scoping reviews. JBI Evid Synth . 2020;18(10):2119–26. Available from: https://journals.lww.com/jbisrir/fulltext/2020/10000/updated_methodological_guidance_for_the_conduct_of.4.aspx. Cited 2025 Jul 22. 10.11124/JBIES-20-00167 33038124

[16] Tricco AC, Lillie E, Zarin W, O’Brien KK, Colquhoun H, Levac D, et al. PRISMA extension for scoping reviews (PRISMA-ScR): Checklist and explanation. Ann Intern Med . 2018;169(7):467–73. Available from: 10.7326/M18-0850. Cited 2025 Jul 22. 10.7326/M18-0850 30178033

[17] Ouzzani M, Hammady H, Fedorowicz Z, Elmagarmid A. Rayyan-a web and mobile app for systematic reviews. Syst Rev . 2016;5(1):1–10. Available from: https://systematicreviewsjournal.biomedcentral.com/articles/10.1186/s13643-016-0384-4. Cited 2025 Jul 22. 10.1186/s13643-016-0384-4 PMC5139140 27919275

[18] Fabrizio CS, van Liere M, Pelto G. Identifying determinants of effective complementary feeding behaviour change interventions in developing countries. Matern Child Nutr. 2014;10(4):575–92. 24798264 10.1111/mcn.12119 PMC4282339

[19] Nayak DS, Kondagunta N, Kamath VG, Kamath A, Nair S. Impact of family level counselling on breast feeding practices and weight gain: A community based cluster randomized controlled trial. Int J Community Med Public Health. 2016;3:486–93.

[20] Nale T, Chavan MK, Mahajan H, Mahajan A. Primary care based interventions are associated with improvement in nutritional status of children: evidence from community based study in India. Int J Scientific Res Public. 2013;3(2):1–5.

[21] Kiran KA, Kujur M, Kumari R, Sagar V, Kumar D, Hassen G, et al. Evaluation of the Health and Nutritional Status of Discharged Children From Malnutrition Treatment Centres Using Mobile Phone Calls During the COVID-19 Lockdown in Jharkhand, India. Cureus . 2023;15(4). Available from: https://pubmed.ncbi.nlm.nih.gov/37261146/. Cited 2025 Jun 10. 10.7759/cureus.38314 PMC10226897 37261146

[22] Adhikari T, Gulati BK, Juneja A, Nair S, Rao MVV, Sharma RK, et al. Development of behaviour change communication model for improving male participation in maternal and child health services among Saharia Tribes in Gwalior district of Madhya Pradesh: a mixed method approach. Int J Community Med Public Health. 2020;7(12):5134.

[23] Panda P, Sahoo T, Parida DD, Bishoi PK, Nayak D, Barad H. Effectiveness Measurement of Development Interventions Among PVTGs In The Nine PVTG Inhabiting States Of India: An Outcome Analysis . Bhubaneswar: Scheduled Castes & Scheduled Tribes Research and Training Institute (SCSTRTI …; 2015. Available from: https://repository.tribal.gov.in/bitstream/123456789/74423/1/SCST_2015_research_0341.pdf. Cited 2025 Dec 3.

[24] Abdalla S, Pair E, Mehta K, Ward V, Mahapatra T, Darmstadt GL. Improving the precision of maternal, newborn, and child health impact through geospatial analysis of the association of contextual and programmatic factors with health trends in Bihar, India. J Glob Health . 2022;12:04064. Available from: https://pubmed.ncbi.nlm.nih.gov/36412069/10.7189/jogh.12.04064 PMC9679706 36412069

[25] Stiller CK. Baseline assessment and effect of a supplementary community-based nutrition intervention study on the prevention/treatment of anemia among young Adivasi children in West Bengal. India: Institute of Nutritional Sciences; 2020.

[26] Armes S, Bhanjdeo A, Chakraborty D, Kaur H, Ray S, Rao N. Aligning Santal Tribe Menu Templates with EAT-Lancet Commission’s Dietary Guidelines for Sustainable and Healthy Diets: A Comparative Analysis. Nutrients . 2024;16(3). Available from: https://pubmed.ncbi.nlm.nih.gov/38337731/. Cited 2025 Jun 10. 10.3390/nu16030447 PMC10856898 38337731

[27] Suresh D. Tribal Development Through Five Year Plans in India–An Overview. The Dawn Journal. 2014;3(1):794–816.

[28] Dutta A. Recommendations of Balanced Nutrition Using Local Food Sourcing for Santhal Tribal Communities. Environ Dev Sustain. 2024;136–46. https://thedawnjournal.in/wp-content/uploads/2013/12/11-Dr.-Devath-Suresh.pdf?srsltid=AfmBOooNwVOW1yDSBqohzC9K1bbtHl-mAbzF_2OfaDHybRXcmgOo82lN.

[29] Bang A. (If) Tribal Children’s Lives Matter, Measure Them! Indian Pediatr. 2021;58(1):11–2. 33452770 10.1007/s13312-021-2349-5 PMC8606277

[30] Kumar A, Rajpal S, Alambusha R, Sharma S, Joe W. Can Anganwadi services strengthening improve the association between maternal and child dietary diversity? Evidence from Project Spotlight implemented in tribal dominated Gadchiroli and Chandrapur districts of Maharashtra, India. PLoS One . 2022;17(3):e0264567. Available from: https://pubmed.ncbi.nlm.nih.gov/35239688/10.1371/journal.pone.0264567 PMC8893689 35239688

[31] Priyanka Singhal PS, Raghuvanshi RS. Development of module for complementary feeding at rural set up. Food Science Research Journal. 2011;2(2):152–6.

[32] Dwivedi R, Goel AD, Vyas V, Sharma PP, Bhardwaj P, Dixit SG, et al. Going the extra mile: Developing an interactive mobile application for maternal and infant care for tribal birth attendants. J Family Med Prim Care. 2024;13(3):990–6. 38736772 10.4103/jfmpc.jfmpc_1315_23 PMC11086811

[33] Das A, Ghosal J, Khuntia HK, Dixit S, Pati S, Kaur H, et al. Malnutrition and Anemia Among Particularly Vulnerable Tribal Groups of Odisha, India: Needs for Context-Specific Intervention. Indian Journal of Community Medicine. 2024;49(3):519–28. 38933794 10.4103/ijcm.ijcm_452_23 PMC11198524

[34] Tandon BN. Nutritional interventions through primary health care: impact of the ICDS projects in India. Bull World Health Organ. 1989;67(1):77–80. 2706729 PMC2491220

[35] Lakhanpaul M, Roy S, Lall MC, Chaturvedi H, Khanna R, Allaham S, et al. Role of schools in community mobilisation to improve IYCF practices in 6-24-month-old tribal children in the Banswara district, India: findings from the qualitative PANChSHEEEL study. BMJ Open. 2022;12(4):e047741. 35414538 10.1136/bmjopen-2020-047741 PMC9006840

[36] Lakhanpaul M, Lall M, Parikh P, Benton L, Dasgupta R, Vijay VK, et al. The PANChSHEEEL Formative report: An integrated health, education, engineering and environmental (HEEE) intervention to optimise infant feeding practices through schools and Anganwadi networks in India. UCL Great Ormond Street Institute of Child Health; 2020. https://discovery.ucl.ac.uk/id/eprint/10137984/1/PANChSHEEEL%20Formative%20Report.pdf.

[37] Avula R, Oddo VM, Kadiyala S, Menon P. Scaling‐up interventions to improve infant and young child feeding in India: what will it take? Matern Child Nutr. 2017;13:e12414. 29032618 10.1111/mcn.12414 PMC6866129

[38] Anant P, Singh PV, Bergkvist S, Haseltine WA, George A. Improving the Health of Mother and Child-Solutions from India . New York; 2012. Available from: https://www.researchgate.net/publication/257931261_Improving_the_Health_of_Mother_and_Child-Solutions_from_India. Cited 2025 Jun 11.

[39] Ministry of Health and Family Welfare: Government of India. Directory of Innovations Implemented in the Health Sector . 2008. Available from: https://nhm.gov.in/WriteReadData/l892s/28174836211472464435.pdf. Cited 2025 Jun 23.

[40] Bang AT, Bang RA, Baitule SB, Reddy MH, Deshmukh MD. Effect of home-based neonatal care and management of sepsis on neonatal mortality: Field trial in rural India. Lancet . 1999;354(9194):1955–61. Available from: https://pubmed.ncbi.nlm.nih.gov/10622298/. Cited 2025 Jun 11. 10.1016/S0140-6736(99)03046-9 10622298

[41] Vir SC, Kalita A, Mondal S, Malik R. Impact of community-based mitanin programme on undernutrition in rural Chhattisgarh State, India. Food Nutr Bull. 2014;35(1):83–91. 24791582 10.1177/156482651403500110

[42] Lakhanpaul M, Sharma S, Roy S, Santwani N, Prakash Pattanaik S, Dang P, et al. Participatory Approach for Nutrition in Children Strengthening Health Education Engineering and Environment Linkages. Available from: https://www.york.ac.uk/media/future-health/events/PANChSHEEEL%20Brochure.pdf. Cited 2025 Dec 3.

[43] Patel A, Kuhite P, Puranik A, Khan SS, Borkar J, Dhande L. Effectiveness of weekly cell phone counselling calls and daily text messages to improve breastfeeding indicators. BMC Pediatr. 2018;18(1). 10.1186/s12887-018-1308-3. 10.1186/s12887-018-1308-3 PMC6206669 30376823

[44] Ministry of Women and Child Development & National Institute of Public Cooperation and Child Development. Potential Good Practices – the ICDS Experience. 2013. https://www.scribd.com/document/518154703/Best-Practices.

[45] Joe W, Subramanyam M, Joshi A, Sharma A, Thube N, Sharma S, et al. Evaluation of ICDS Scheme of India. 2020. https://www.niti.gov.in/sites/default/files/2023-03/Evaluation%20of%20ICDS%20Scheme%20of%20India.pdf.

[46] Mustaphi P, Dobe M. Positive deviance--the West Bengal experience. Indian J Public Health. 2005;49(4):207–13. 16479899

[47] Sharma S, Krishnan A, Pradhan R, Sadanshiv AS, Pardhee A, Joe W, et al. Project Spotlight: Implementation strategy Integrated Child Development Services System Strengthening and Community Mobilization Initiative in Maharashtra . Mumbai, India; 2021. Available from: https://www.tatatrusts.org/Upload/Content_Files/implementation-strategy-project-spotlight.pdf. Cited 2025 Jun 11.

[48] IntraHealth International. The Vistaar Project Project Close-Out Report . 2013. Available from: https://www.intrahealth.org/sites/default/files/attachment-files/vistaar-project-close-out-report.pdf. Cited 2025 Jun 20.

[49] Vazir S, Engle P, Balakrishna N, Griffiths PL, Johnson SL, Creed-Kanashiro H, et al. Cluster-randomized trial on complementary and responsive feeding education to caregivers found improved dietary intake, growth and development among rural Indian toddlers. Matern Child Nutr. 2013;9(1):99–117. 22625182 10.1111/j.1740-8709.2012.00413.x PMC3434308

[50] Madhumathi A. The Effect of Developed Educational Material on the Knowledge Content of Tribal Mothers in Child Care . Andhra Pradesh Agricultural University; 1993. Available from: http://krishikosh.egranth.ac.in/handle/1/5810004935

[51] Kilaru A, Griffiths PL, Ganapathy S, Shanti G. Community-based nutrition education for improving infant growth in rural Karnataka. Indian Pediatr. 2005;42(5):425–32. 15923688

[52] Deepa M. Effect of Intervention on Developmental status of Children and Health and Nutritional Status of Tribal Families. College of Home Science, Professor Jayashankar Telangana State Agricultural University. 2017. https://krishikosh.egranth.ac.in/bitstreams/456e60e9-0172-4fef-9ea7-85a9b17da826/download.

[53] Mondal S, Joe W, Akhauri S, Thakur P, Kumar A, Pradhan N, et al. Association of BCC module roll-out in SHG meetings with changes in complementary feeding and dietary diversity among children (6–23 months)? Evidence from JEEViKA in rural Bihar, India. PLoS ONE. 2023;18(1):e0279724. 36602987 10.1371/journal.pone.0279724 PMC9815627

[54] Saggurti N, Atmavilas Y, Porwal A, Schooley J, Das R, K, et al. Effect of health intervention integration within women’s self-help groups on collectivization and healthy practices around reproductive, maternal, neonatal and child health in rural India. PLoS One. 2018;13(8):e0202562. 10.1371/journal.pone.0202562 PMC6107172 30138397

[55] Husain Z, Dutta M. Impact of Self Help Group membership on the adoption of child nutritional practices: evidence from JEEViKA’s health and nutrition strategy programme in Bihar, India. J Int Dev. 2023;35(5):781–99.

[56] Kumar S. Improving breastfeeding practices through community based organizations-evidences from care’s child survival project in Jharkhand, India. J Neonatol. 2004;18(2):53–65.

[57] Kadiyala S, Harris-Fry H, Pradhan R, Mohanty S, Padhan S, Rath S, et al. Effect of nutrition-sensitive agriculture interventions with participatory videos and women’s group meetings on maternal and child nutritional outcomes in rural Odisha, India (UPAVAN trial): a four-arm, observer-blind, cluster-randomised controlled trial. Lancet Planet Health . 2021;5(5):e263–76. Available from: https://pubmed.ncbi.nlm.nih.gov/33811818/. Cited 2025 May 8. 10.1016/S2542-5196(21)00001-2 PMC8099729 33811818

[58] Banerjee B, Mandal ON. An intervention study in malnutrition among infants in a tribal community of West Bengal. Indian journal of community medicine. 2005;30(1):27.

[59] Chaudhuri SN. CINI’s approaches to intervention: an innovative strategy to combat malnutrition in India. Nutr Rev. 2002;60(5 Pt 2):S102–8. 12035846 10.1301/00296640260130830

[60] Bhowmick S, Ghosh D. Evaluation of the impact of awareness intervention in promoting breastfeeding and child-feeding among tribal mothers of under-5 children in Jangalmahal region of West Bengal: Role of Theory of Planned Behavior. J Indian Anthropol Soc. 2023;58(2).

[61] Raghunathan K, Alvi M, Sehgal M. Ethnicity, information and cooperation: evidence from a group-based nutrition intervention. Food Policy. 2023;120:102478. 38028948 10.1016/j.foodpol.2023.102478 PMC10679797

[62] Surve S, Kulkarni R, Patil S, Sankhe L. Impact of intervention on nutritional status of under-fives in tribal blocks of Palghar District in Maharashtra, India. Indian J Public Health . 2022;66(2):159–65. Available from: https://pubmed.ncbi.nlm.nih.gov/35859498/10.4103/ijph.ijph_1770_21 35859498

[63] Srivastava A, Gwande K, Bhattacharya S, Singh VK. Impact of the positive deviance approach on breastfeeding practices among tribal pregnant women: a before–after intervention study. CHRISMED J Health Res. 2019;6(4):222–8.

[64] Desai L, Shah P, Shah S, Vani SN. Integrated interventions to reduce neonatal mortality in a tribal area of South Gujarat. J Neonatol. 2005;19(1):21–9.

[65] Seksaria SA, Sheth MK. Mass media campaign to improve infant and young child feeding practices amongst tribal mothers of Chikhli taluka, Gujarat. American International Journal of Research in Humanities, Arts and Social Sciences. 2014;191–5.

[66] Modi D, Dholakia N, Gopalan R, Venkatraman S, Dave K, Shah S, et al. mHealth intervention “ImTeCHO” to improve delivery of maternal, neonatal, and child care services-A cluster-randomized trial in tribal areas of Gujarat, India. PLoS Med . 2019;16(10):e1002939. Available from: https://pubmed.ncbi.nlm.nih.gov/31647821/10.1371/journal.pmed.1002939 PMC6812744 31647821

[67] Hazra A, Atmavilas Y, Hay K, Saggurti N, Verma RK, Ahmad J, et al. Effects of health behaviour change intervention through women’s self-help groups on maternal and newborn health practices and related inequalities in rural India: a quasi-experimental study. EClinMed. 2020. 10.1016/j.eclinm.2019.10.011. 10.1016/j.eclinm.2019.10.011 PMC6978187 31993574

[68] Singh V, Ahmed S, Dreyfuss ML, Kiran U, Chaudhery DN, Srivastava VK, et al. Non-governmental organization facilitation of a community-based nutrition and health program: effect on program exposure and associated infant feeding practices in rural India. PLoS ONE. 2017;12(9):e0183316. 28910328 10.1371/journal.pone.0183316 PMC5598933

[69] Tripathy P, Nair N, Barnett S, Mahapatra R, Borghi J, Rath S, et al. Effect of a participatory intervention with women’s groups on birth outcomes and maternal depression in Jharkhand and Orissa, India: a cluster-randomised controlled trial. Lancet. 2010;375(9721):1182–92. 20207411 10.1016/S0140-6736(09)62042-0

[70] Nair N, Tripathy P, Sachdev HS, Pradhan H, Bhattacharyya S, Gope R, et al. Effect of participatory women’s groups and counselling through home visits on children’s linear growth in rural eastern India (CARING trial): a cluster-randomised controlled trial. Lancet Glob Health. 2017;5(10):e1004–16. 28911749 10.1016/S2214-109X(17)30339-X PMC5640793

[71] Saxton JC. Assessing the potential of community mobilisation with women’s groups to improve child growth among underserved tribal communities of Eastern India. The UCL Institute of Global Health; 2013. https://discovery.ucl.ac.uk/id/eprint/1400637/.

[72] Gope RK, Tripathy P, Prasad V, Pradhan H, Sinha RK, Panda R, et al. Effects of participatory learning and action with women’s groups, counselling through home visits and crèches on undernutrition among children under three years in eastern India: a quasi-experimental study. BMC Public Health. 2019;19(1):962. 31319828 10.1186/s12889-019-7274-3 PMC6637592

[73] Rasaily R, Ganguly KK, Roy M, Vani SN, Kharood N, Kulkarni R, et al. Community based kangaroo mother care for low birth weight babies: a pilot study. Indian J Med Res. 2017;145(1):51–7. 10.4103/ijmr.IJMR_603_15. 28574014 10.4103/ijmr.IJMR_603_15 PMC5460573

[74] Scott S, Gupta S, Menon P, Raghunathan K, Thai G, Quisumbing A, et al. A quasi-experimental evaluation of a nutrition behavior change intervention delivered through women’s self-help groups in rural India: impacts on maternal and young child diets, anthropometry, and intermediate outcomes. Curr Dev Nutr. 2022;6(6):nzac079. 35694241 10.1093/cdn/nzac079 PMC9177383

[75] Gope RK, Tripathy P, Prasad V, ana, Pradhan H, Sinha RK, et al. Effects of participatory learning and action with women’s groups, counselling through home visits and crèches on undernutrition among children under three years in eastern India: a quasi-experimental study. BMC Public Health. 2019;19(1):962. 10.1186/s12889-019-7274-3 PMC6637592 31319828

[76] Sharma M, Gaidhane A, Choudhari SG. A Review of Infant and Young Child Feeding Practices and Their Challenges in India. Cureus . 16(8):e66499. Available from: https://www.ncbi.nlm.nih.gov/pmc/articles/PMC11381101/10.7759/cureus.66499 PMC11381101 39246879

[77] Wanjohi M, Griffiths P, Wekesah F, Muriuki P, Muhia N, Musoke RN, et al. Sociocultural factors influencing breastfeeding practices in two slums in Nairobi, Kenya. Int Breastfeed J . 2017;12(1):1–8. Available from: https://link.springer.com/articles/10.1186/s13006-016-0092-7. Cited 2025 Jun 25. 10.1186/s13006-016-0092-7 PMC5225512 28096888

[78] Chakona G. Social circumstances and cultural beliefs influence maternal nutrition, breastfeeding and child feeding practices in South Africa. Nutr J . 2020;19(1):1–15. Available from: https://nutritionj.biomedcentral.com/articles/10.1186/s12937-020-00566-4. Cited 2025 Jun 25. 10.1186/s12937-020-00566-4 PMC7240933 32434557

[79] Ministry of Tribal Affairs. Statewise Total & Tribal Population of India (As per 2011 Census) . Available from: https://trti.maharashtra.gov.in/statewiseTotalTribal. Cited 2025 Jun 13.

[80] Tribal Welfare Department: Government of Telangana. Tribes of Telangana. Available from: https://forestrights.telangana.gov.in/Website/Tribes.aspx. Cited 2025 Jun 13.

[81] Islary J. Problems and Challenges of Researching Tribal Health in India. Journal of Tribal Intellectual Collective India . 2024;7(10):81–90. Available from: https://www.ticijournals.org/problems-and-challenges-of-researching-tribal-health-in-india/. Cited 2025 Jul 22.

[82] M R, Shabadi N, Kulkarni P, Sunil Kumar D, Anup G, Narayana Murthy MR. Effectiveness of educational intervention on breastfeeding among primi pregnant women- a longitudinal study. Clin Epidemiol Glob Health . 2020;8(4):1306–11. Available from: https://www.sciencedirect.com/science/article/pii/S2213398420301287. Cited 2025 Jun 13.

[83] Boynito WG, Diongue O, Temesgen K, Yeshitila YG, Tessema GY, De Souza M, et al. Effectiveness of video-based health education on breastfeeding practices among infants aged 0–6 months in Dirashe District, South Ethiopia: a cluster randomized controlled trial. Hum Behav Emerg Technol. 2024;2024(1):2158432. 10.1155/2024/2158432.

[84] Piro SS, Ahmed HM. Impacts of antenatal nursing interventions on mothers’ breastfeeding self-efficacy: An experimental study. BMC Pregnancy Childbirth . 2020;20(1):1–12. Available from:https://bmcpregnancychildbirth.biomedcentral.com/articles/10.1186/s12884-019-2701-0. Cited 2025 Jun 13. 10.1186/s12884-019-2701-0 PMC6945460 31906881

[85] Mal P, Saikia N. Empowering tribal women: a comparative analysis of matrilineal and patrilineal societies in India. Cogent Soc Sci . 2024;10(1):2360172. Available from: https://www.tandfonline.com/doi/pdf/10.1080/23311886.2024.2360172. Cited 2025 Jun 25.

[86] Shrestha S, Pokhrel M, Mathema S. Knowledge, Attitude and Practices among Mothers of Children 6 to 24 months of Age Regarding Complementary Feeding. JNMA J Nepal Med Assoc . 2020;58(230):758. Available from: https://pmc.ncbi.nlm.nih.gov/articles/PMC7654499/. Cited 2025 Dec 2. 10.31729/jnma.5274 PMC7654499 34504365

[87] Şişko SG, Bağ Ö, Kondolot M, Nalbantoğlu B, Gökcay G. Breastfeeding and Infant Nutrition Knowledge, Attitude, and Practices of Parents. Turkish archives of pediatrics . 2022;57(4):441–7. Available from: https://pubmed.ncbi.nlm.nih.gov/35822477/. Cited 2025 Dec 2. 10.5152/TurkArchPediatr.2022.21201 PMC9317703 35822477

[88] Bekele T, Rawstorne P, Rahman B. Effect of water, sanitation and hygiene interventions alone and combined with nutrition on child growth in low and middle income countries: a systematic review and meta-analysis. BMJ Open . 2020;10(7):e034812. Available from: https://pmc.ncbi.nlm.nih.gov/articles/PMC7359184/. Cited 2025 Jun 16. 10.1136/bmjopen-2019-034812 PMC7359184 32660947

[89] Has EMM, Nursalam N, Arief YS. Women’s Empowerment and Infant and Young Child Feeding Practice in Low- and Middle-Income Countries: A Systematic Review. Proceedings of the 4th International Conference on Sustainable Innovation 2020–Health Science and Nursing (ICoSIHSN 2020). 2021;33. https://www.atlantis-press.com/proceedings/icosihsn-20/125951226.

[90] Raj A, Ghule M, Ritter J, Battala M, Gajanan V, Nair S, et al. Cluster Randomized Controlled Trial Evaluation of a Gender Equity and Family Planning Intervention for Married Men and Couples in Rural India. PLoS One . 2016;11(5). Available from: https://pubmed.ncbi.nlm.nih.gov/27167981/. Cited 2025 Dec 2. 10.1371/journal.pone.0153190 PMC4864357 27167981

[91] Jungari S, Paswan B. Male perception and participation in family planning among tribal communities of Maharashtra, India: a mixed-method study. Int Q Community Health Educ. 2020;40(3):163–9. 10.1177/0272684X19875017. 31490740 10.1177/0272684X19875017

[92] Parija PP, Pal A, Panigrahi SK, Thakur P, Pal R. Male involvement in family planning in a rural area of India. J Family Med Prim Care . 2022;11(5):1943. Available from: https://pmc.ncbi.nlm.nih.gov/articles/PMC9254820/. Cited 2025 Dec 2. 10.4103/jfmpc.jfmpc_1557_21 PMC9254820 35800530

[93] Diamond-Smith N, Vaishnav Y, Choudhary U, Sharma P, Kachhwaha A, Panjalingam T, et al. Individual empowerment and community norm effects of engaging young husbands in reproductive health in rural India: findings from a pilot study. Reprod Health . 2024;21(1):147. Available from: https://pmc.ncbi.nlm.nih.gov/articles/PMC11488357/. Cited 2025 Dec 2. 10.1186/s12978-024-01878-y PMC11488357 39420379

[94] Chatterjee S. Stigma, judgment, and misinformation: The bumpy road to sexual reproductive health in India - The Hindu. 2025; Available from: https://www.thehindu.com/sci-tech/health/stigma-judgment-and-misinformation-the-bumpy-road-to-sexual-reproductive-health-in-india/article69206109.ece. Cited 2025 Dec 2.

[95] Jose JP, Cherayi SJ, Raju KT. A Qualitative Enquiry into the Tribal Mothers’ Breastfeeding and Related Hygiene Practices in Kerala. Indian Journal of Health and Wellbeing. 2020;11(01).

[96] Maleki-Saghooni N, Amel Barez M, Karimi FZ. Investigation of the relationship between social support and breastfeeding self-efficacy in primiparous breastfeeding mothers. Journal of Maternal-Fetal and Neonatal Medicine . 2020;33(18):3097–102. Available from: https://www.tandfonline.com/doi/pdf/10.1080/14767058.2019.1568986. Cited 2025 Jun 13. 10.1080/14767058.2019.1568986 30632820

[97] Sarkar D, Dalai CK, Sarkar K, Das SS, Banerjee S. Breastfeeding practices and infant feeding pattern of a tribal population region of eastern India. J Family Med Prim Care . 2020;9(9):4570. Available from: https://pmc.ncbi.nlm.nih.gov/articles/PMC7652134/. Cited 2025 Jun 25. 10.4103/jfmpc.jfmpc_631_20 PMC7652134 33209765

[98] Schneider L, Korhonen K, Ollila S, Mutanen M. Social realities in remote villages: Infant and young child feeding in Kirewa, Uganda. PLOS Global Public Health . 2024;4(9):e0003016. Available from: https://pmc.ncbi.nlm.nih.gov/articles/PMC11386423/. Cited 2025 Dec 2. 10.1371/journal.pgph.0003016 PMC11386423 39255291

[99] Lopes CVA, de Sousa Alves Neri JL, Hunter J, Ronto R, Mihrshahi S. Interventions and Programs Using Native Foods to Promote Health: A Scoping Review. Nutrients . 2024;16(23):4222. Available from: https://pmc.ncbi.nlm.nih.gov/articles/PMC11644025/. Cited 2025 Jun 13. 10.3390/nu16234222 PMC11644025 39683615

[100] Shakya P, Kunieda MK, Koyama M, Rai SS, Miyaguchi M, Dhakal S, et al. Effectiveness of community-based peer support for mothers to improve their breastfeeding practices: A systematic review and meta-analysis. PLoS One . 2017;12(5):e0177434. Available from: https://pmc.ncbi.nlm.nih.gov/articles/PMC5433692/. Cited 2025 Jun 13. 10.1371/journal.pone.0177434 PMC5433692 28510603

[101] Ara G, Khanam M, Papri N, Nahar B, Kabir I, Sanin KI, et al. Peer Counseling Promotes Appropriate Infant Feeding Practices and Improves Infant Growth and Development in an Urban Slum in Bangladesh: A Community-Based Cluster Randomized Controlled Trial. Curr Dev Nutr . 2019;3(7):nzz072. Available from: https://pmc.ncbi.nlm.nih.gov/articles/PMC6635820/. Cited 2025 Jun 13. 10.1093/cdn/nzz072 PMC6635820 31334480

[102] Saha A, Kasi E. Mapping Self-Help Groups (SHGs) as alternatives to capitalist development: an ethnographic enquiry from India. Sustain Sci . 2022;17(4):1263–71. Available from: https://link.springer.com/article/10.1007/s11625-022-01171-6. Cited 2025 Jun 13.

[103] Verma A, Nguyen T, Purty A, Pradhan N, Husan A, Zambrano P, et al. Changing maternal and child nutrition practices through integrating social and behavior change interventions in community-based self-help and support groups: literature review from Bangladesh, India, and Vietnam. Front Nutr . 2024;11:1464822. Available from: https://pmc.ncbi.nlm.nih.gov/articles/PMC11602304/. Cited 2025 Jun 13. 10.3389/fnut.2024.1464822 PMC11602304 39610881

[104] Younes L, Houweling TAJ, Azad K, Kuddus A, Shaha S, Haq B, et al. The effect of participatory women’s groups on infant feeding and child health knowledge, behaviour and outcomes in rural Bangladesh: a controlled before-and-after study. J Epidemiol Community Health (1978) . 2014;69(4):374. Available from: https://pmc.ncbi.nlm.nih.gov/articles/PMC4392217/. Cited 2025 Jun 13. 10.1136/jech-2014-204271 PMC4392217 25472635

[105] Arriola-Pacheco F, Ness A, Sihuay-Torres K, Garcia-Quintana A, Proaño D, Lawrence HP. Community-Based Participatory Research: Lessons and Challenges. Symposium Special Communication. JDR Clin Trans Res . 2024;10(2):96. Available from: https://pmc.ncbi.nlm.nih.gov/articles/PMC11894848/. Cited 2025 Dec 2. 10.1177/23800844241266505 PMC11894848 39279248

[106] Chaudhary I, Ranjan R. Opinion: Contextualising Initiatives For Transforming Nutritional Outcomes Of Tribal Communities. NDTV Swachh India . 2023; Available from: https://swachhindia.ndtv.com/opinion-contextualising-initiatives-for-transforming-nutritional-outcomes-of-tribal-communities-83404/. Cited 2025 May 10.

[107] The George Institute for Global Health. The George Institute India releases study on contribution of indigenous foods to address malnutrition in Vulnerable Tribal Communities in India . 2023. Available from: https://georgeinstitute.org/news-and-media/news/the-george-institute-india-releases-study-on-contribution-of-indigenous-foods-to-address. Cited 2025 May 10.

[108] Sharma N, Gupta M, Aggarwal AK, Gorle M. Effectiveness of a culturally appropriate nutrition educational intervention delivered through health services to improve growth and complementary feeding of infants: A quasi-experimental study from Chandigarh, India. PLoS One . 2020;15(3):e0229755. Available from: https://pmc.ncbi.nlm.nih.gov/articles/PMC7077818/. Cited 2025 Jun 16. 10.1371/journal.pone.0229755 PMC7077818 32182241

[109] Livingstone KM, Love P, Mathers JC, Kirkpatrick SI, Olstad DL. Cultural adaptations and tailoring of public health nutrition interventions in Indigenous peoples and ethnic minority groups: opportunities for personalised and precision nutrition. Proc Nutr Soc. 2023;82(4):478–86. 10.1017/S002966512300304X. 37334485 10.1017/S002966512300304X

[110] Kim SS, Nguyen PH, Yohannes Y, Abebe Y, Tharaney M, Drummond E, et al. Behavior Change Interventions Delivered through Interpersonal Communication, Agricultural Activities, Community Mobilization, and Mass Media Increase Complementary Feeding Practices and Reduce Child Stunting in Ethiopia. J Nutr . 2019;149(8):1470. Available from: https://pmc.ncbi.nlm.nih.gov/articles/PMC6686053/. Cited 2025 Jun 16. 10.1093/jn/nxz087 PMC6686053 31165869

[111] Chakona G. Social circumstances and cultural beliefs influence maternal nutrition, breastfeeding and child feeding practices in South Africa. Nutrition Journal 2020 19:1 . 2020;19(1):47-. Available from: https://link.springer.com/article/10.1186/s12937-020-00566-4. Cited 2025 Nov 29. 10.1186/s12937-020-00566-4 PMC7240933 32434557

[112] Lakhanpaul M, Roy S, Benton L, Lall M, Khanna R, Vijay VK, et al. Why India is struggling to feed their young children? A qualitative analysis for tribal communities. BMJ Open . 2022;12(7):e051558. Available from: https://pubmed.ncbi.nlm.nih.gov/35902199/. 10.1136/bmjopen-2021-051558 PMC9341212 35902199

